# Author Correction: Nonlinearity of root trait relationships and the root economics spectrum

**DOI:** 10.1038/s41467-022-28444-z

**Published:** 2022-02-08

**Authors:** Deliang Kong, Junjian Wang, Huifang Wu, Oscar J. Valverde-Barrantes, Ruili Wang, Hui Zeng, Paul Kardol, Haiyan Zhang, Yulong Feng

**Affiliations:** 1 Liaoning Key Laboratory for Biological Invasions and Global Change, Shenyang Agricultural University, 110866 Shenyang, Liaoning Province China; 2 School of Environmental Science and Engineering, Guangdong Provincial Key Laboratory of Soil and Groundwater Pollution Control, Southern University of Science and Technology, 518055 Shenzhen, China; 3 School of Life Sciences, Henan University, 475004 Kaifeng, China; 4 International Center of Tropical Botany, Florida International University, Miami, FL 33199 USA; 5 College of Forestry, Northwest A&F University, 712100 Yangling, China; 6 Key Laboratory for Urban Habitat Environmental Science and Technology, Peking University Shenzhen Graduate School, 518005 Shenzhen, China; 7 Department of Forest Ecology and Management, Swedish University of Agricultural Sciences, 90183 Umeå, Sweden

**Keywords:** Biogeography, Ecophysiology, Evolutionary ecology, Forest ecology, Biogeography, Ecophysiology, Evolutionary ecology, Forest ecology

Correction to: *Nature Communications* 10.1038/s41467-019-10245-6, published online 17 May 2019.

In the Acknowledgements section of this article, the grant number relating to the National Natural Science Foundation of China given for Deliang Kong was incorrectly given as 31870552 and should have been 31870522. The original article has been corrected.

