# Supplementary material for: Nonlinearity of root trait relationships and the root economics spectrum
Source: Nat Commun. 2019 May 17;10:2203. doi: 10.1038/s41467-019-10245-6 (PMC6525182; doi:10.1038/s41467-019-10245-6)
Supplement: Supplementary file 1 — Supplementary Information [file 41467_2019_10245_MOESM1_ESM.pdf]

Supplementary Information

Nonlinearity of root trait relationships and the root economics spectrum

Deliang Kong[1*], Junjian Wang[2*], Huifang Wu[3], Oscar J. Valverde-Barrantes[4], Ruili Wang[5], Hui Zeng[6], Paul Kardol[7], Haiyan Zhang[1], Yulong Feng[1*]

[1]Liaoning Key Laboratory for Biological Invasions and Global Change, Shenyang Agricultural University, Shenyang, Liaoning Province 110866, China

[2]School of Environmental Science and Engineering, Guangdong Provincial Key Laboratory of Soil and Groundwater Pollution Control, Southern University of Science and Technology, Shenzhen, 518055, China

[3]School of Life Sciences, Henan University, Kaifeng, 475004, China

[4]International Center of Tropical Botany, Florida International University, Miami, FL 33199, USA

[5]College of Forestry, Northwest A&F University, Yangling, 712100, China

[6]Key Laboratory for Urban Habitat Environmental Science and Technology, Peking University Shenzhen Graduate School, Shenzhen, 518005, China

[7]Department of Forest Ecology and Management, Swedish University of Agricultural Sciences, Umeå, 90183, Sweden

D.K., J.W., H.W., O.J.V. and R.W. contributed equally to this study

Authors for correspondence: D.K. email: deliangkong1999@126.com; J.W. email: wangjj@sustech.edu.cn; Y.F. email: yl_feng@tom.com

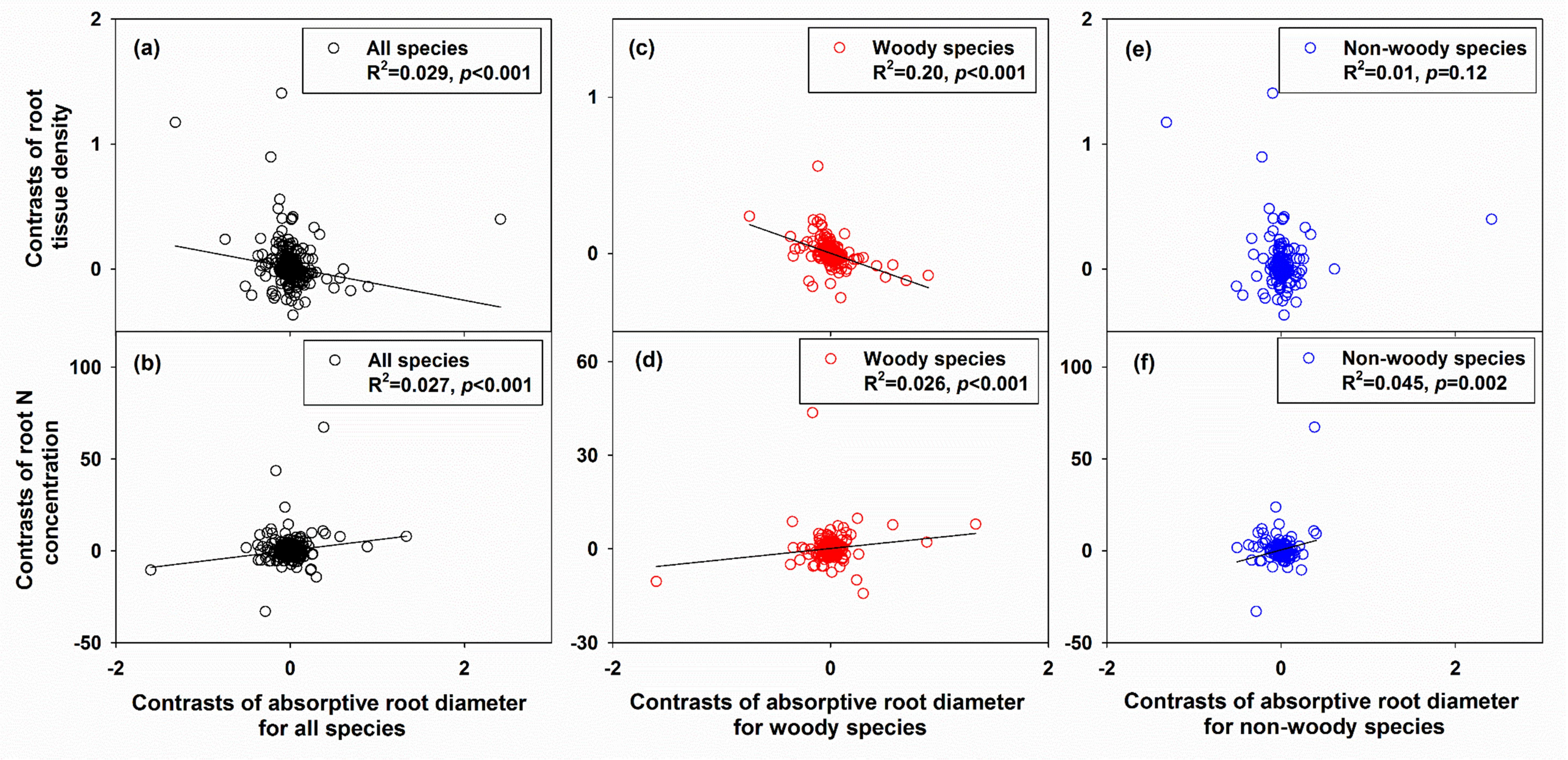

Supplementary Fig. 1 Relationships between absorptive root diameter and root tissue density and root N concentration across all species (a,b), for woody species (c,d) and for non-woody species (e,f) using phylogenetic independent contrasts.

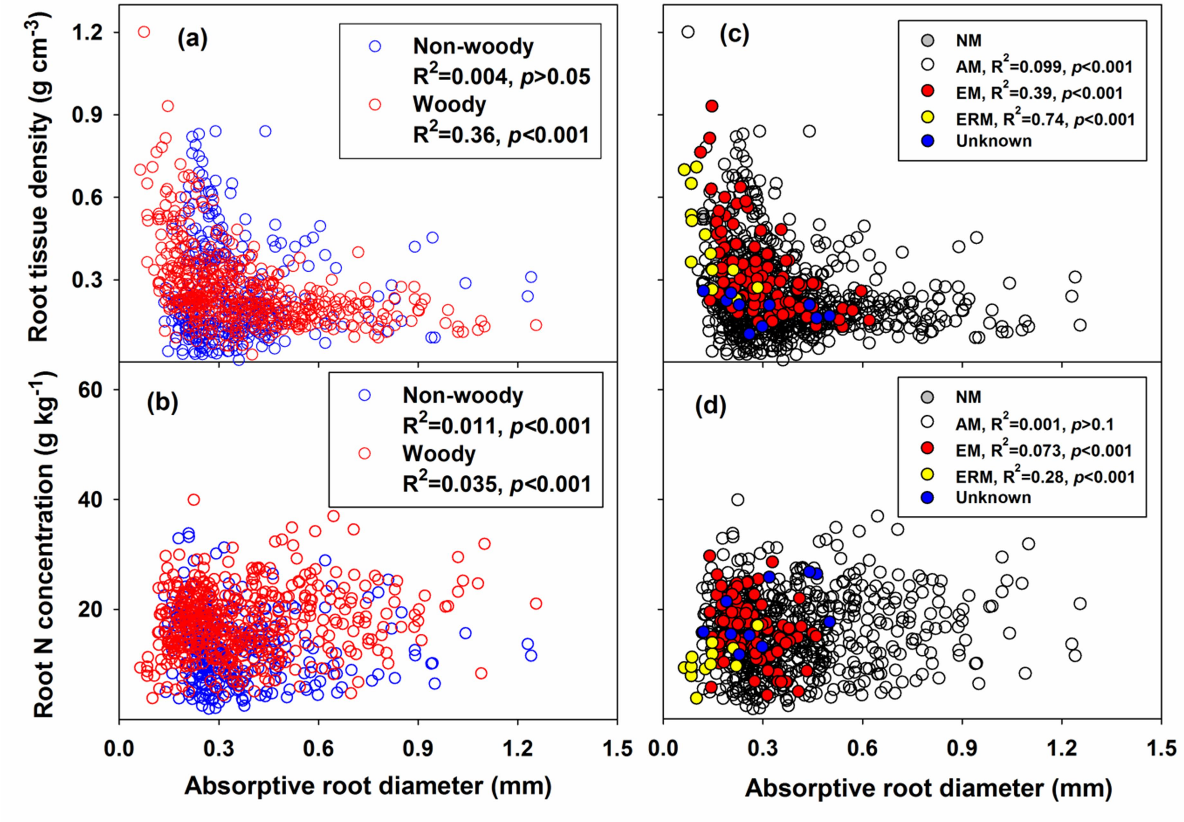

Supplementary Fig. 2 Relationships between root diameter and root tissue density (RTD) and root N concentration (RN) for woody and non-woody species (a,b) and for different mycorrhizal types (NM = non-mycorrhiza; AM = arbuscular mycorrhiza; EM = ectomycorrhiza; ERM = ericoid mycorrhiza; and Unknown = unknown mycorrhizal status) for all species (c,d). See Supplementary Data 2 for the regression equations.

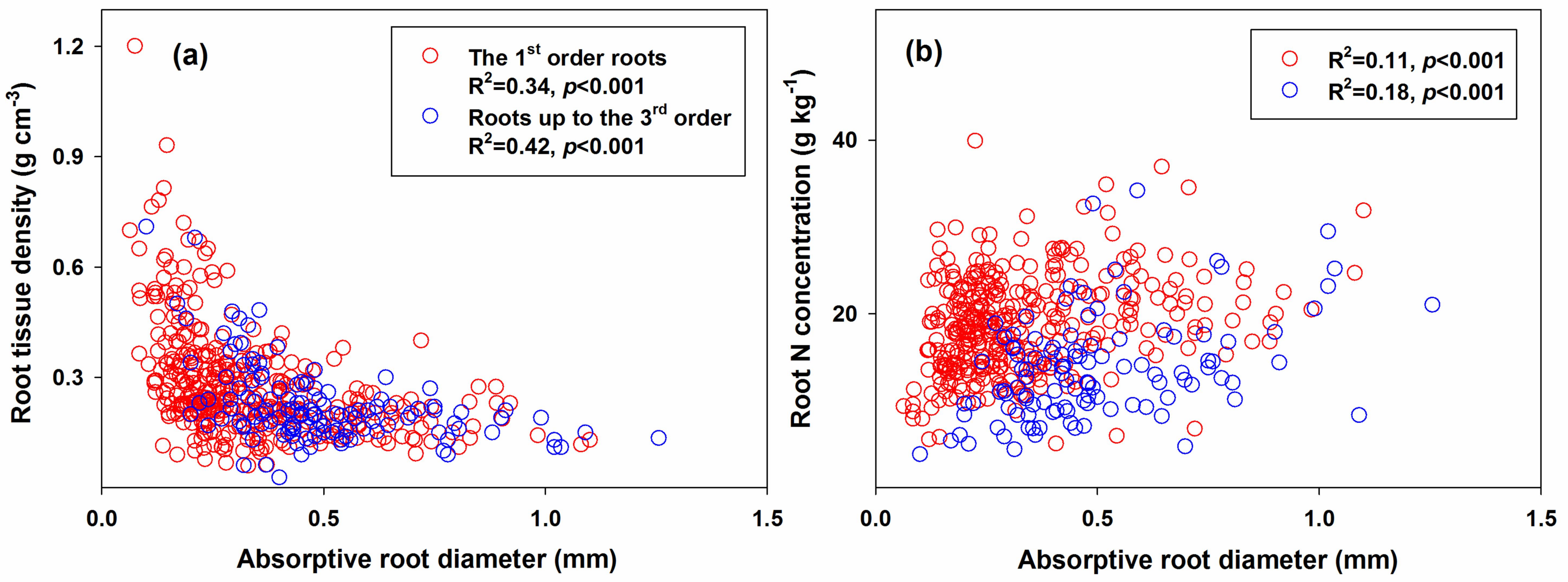

Supplementary Fig. 3 Relationships between absorptive root diameter and root tissue density (a), and root nitrogen concentration (b) in woody species. Studies using the 1st order roots are indicated by red circles, and studies using up to the 3rd order of roots are indicated by blue circles. See Table S1 for the data source and Supplementary Data 2 for the regression equations.

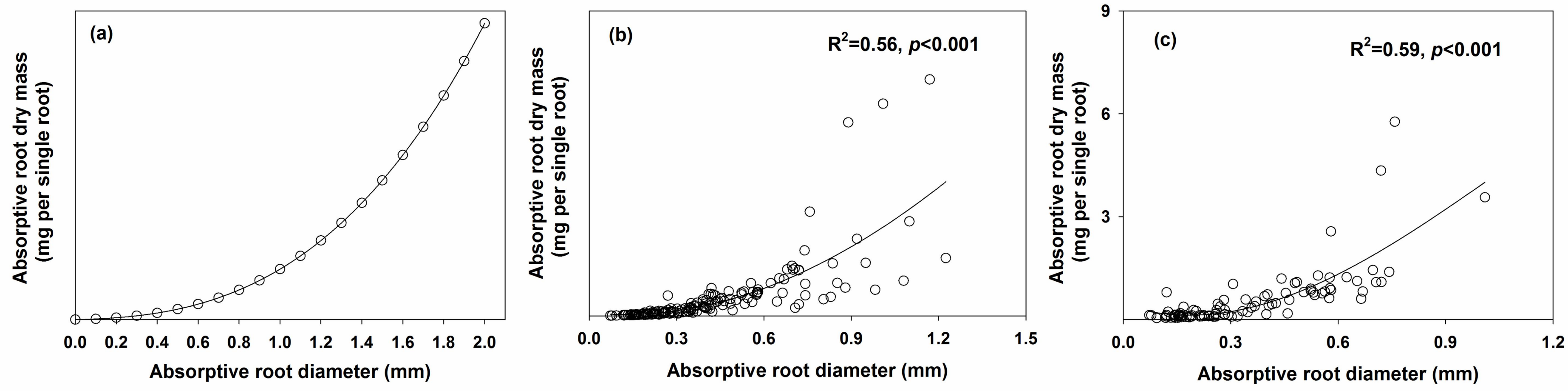

Supplementary Fig. 4 Theoretical relationship between root diameter (x) and root dry mass (y) for absorptive roots using randomly simulated data (a), root anatomical data (b) and measured root mass (c) for woody species. The relationship is based on individual absorptive roots, i.e., the single 1st order roots. In panel (a), root dry mass is expressed as the product of root volume (v) and root tissue density (ρ): y=ρv. Root length (RL) has been shown to be positively related to root diameter[1,2]: RL=k_1x. Root tissue density is proportional to the proportion of root cross-sectional area occupied by the steles (i.e., PRS): $\rho=k_2\times PRS=k_2(0.5\text{-}k\text{-}c/x)^2$, where k=0.43 and c=-0.016 (see Introduction and Supplementary Data 2). Then, root dry mass can be expressed as: $y=\rho v=\pi k_1k_2x^3(0.5\text{-}k\text{-}c/x)^2$. It can also be rearranged to the following cubic function: $y=\pi k_1k_2((0.5\text{-}k)^2x^3\text{-}2c(0.5\text{-}k)x^2+c^2x)$. In panel (a), y is simulated by randomly generating x values from 0 to 2 with an interval of 0.1. In panel (b), we examine the relationship between root dry mass and root diameter using the following function: $y=\rho v= 0.25\pi k_1k_2x^3\times PRS$. Root diameter and PRS in the function are from studies[2,3,4,5] in the Supplementary References. In panel (c), we fit the relationship between the 1st order root diameter and root mass in a cubic regression using data from Kong's study[2] as well as the Source Data for 96 woody species. Source data are provided as a Source Data file.

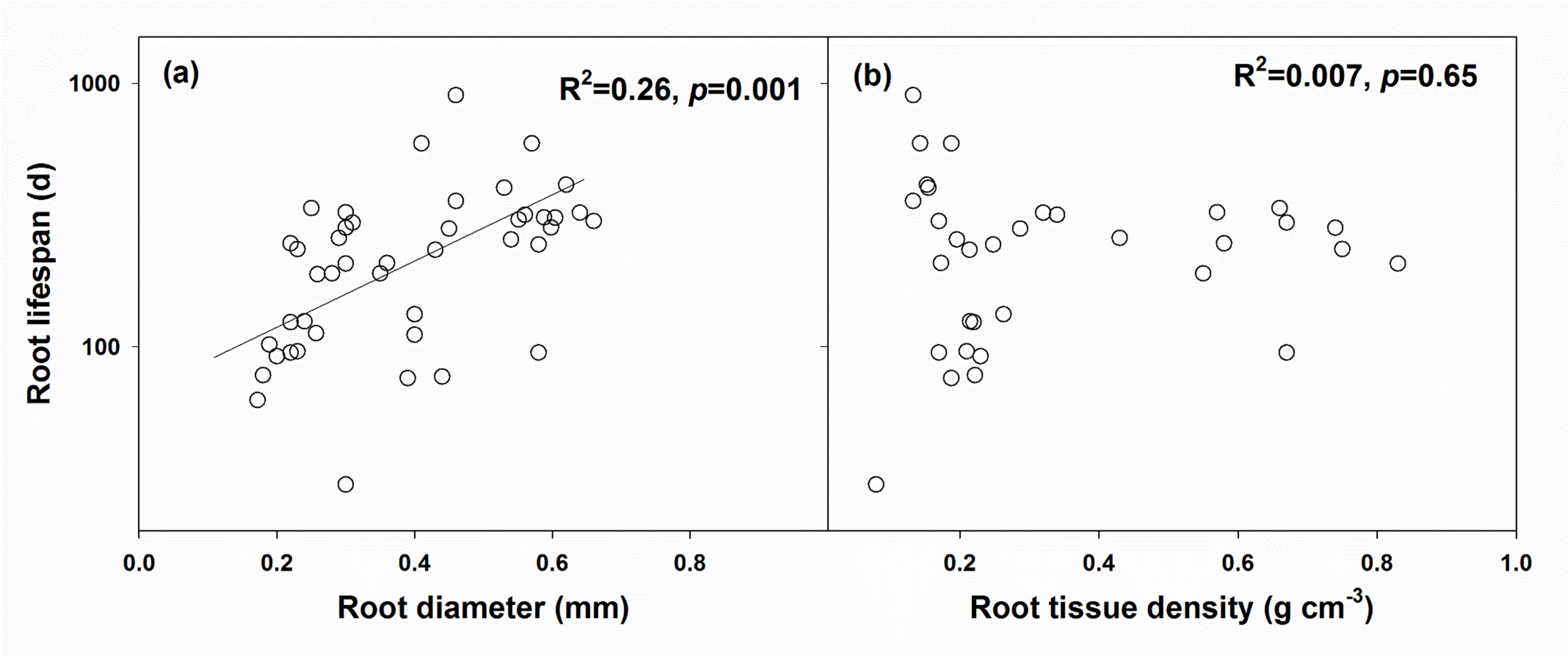

Supplementary Fig. 5 Relationship between root lifespan (log-transformed) and root diameter (a) and root tissue density (b) for absorptive roots of woody species. Data are from studies[4,6,7,8,9,10,11] in the Supplementary References.

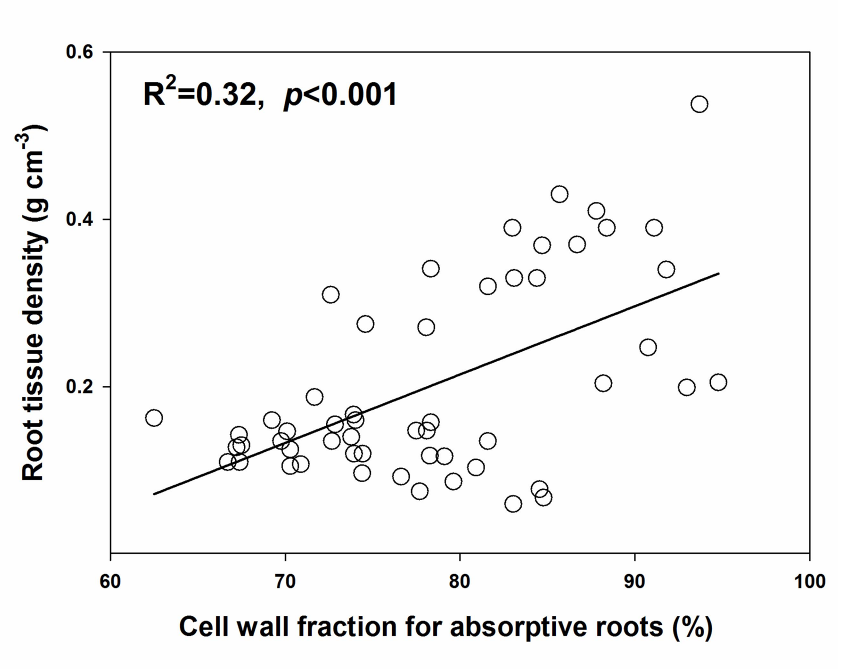

Supplementary Fig. 6 Relationship between root cell wall fraction and root tissue density. Root cell wall fraction is the sum of the acid-soluble fraction and the acid insoluble fraction. Data are from studies[5,12,13,14] in the Supplementary References. These data are largely from woody species.

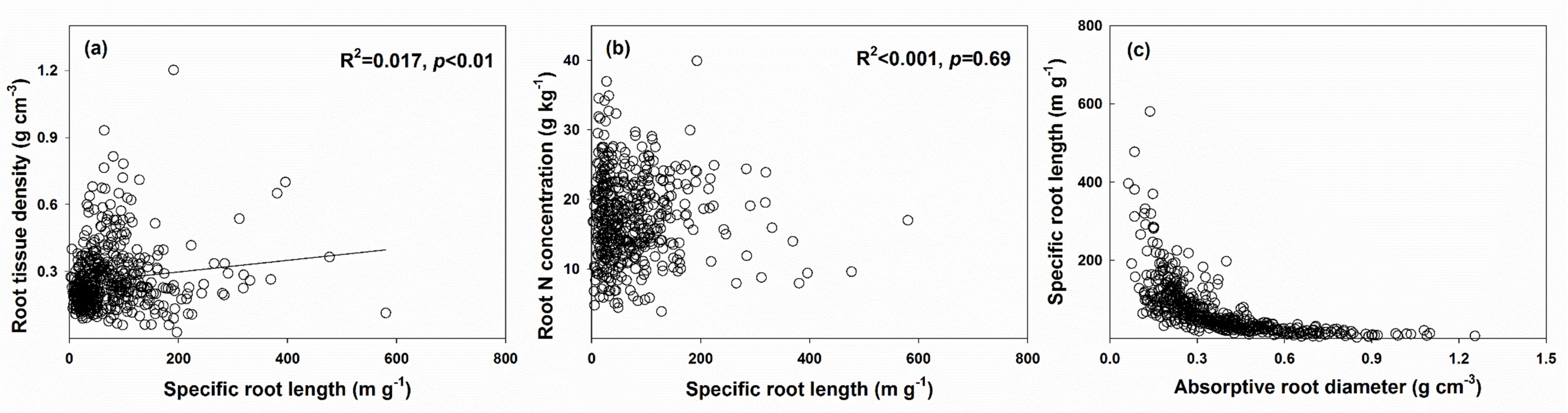

Supplementary Fig. 7 Relationships of specific root length with root tissue density (a), root N concentration (b) and absorptive root diameter (c) for woody species. See Supplementary Data 1 for the data sources.

Supplementary Table 1 Results of phylogenetic generalized least squares and ordinary least squares analyses for the effects of root diameter (Diameter), Growth form (woody vs. non-woody) and mycorrhizal type (AM, EM and ERM) on root tissue density (RTD) and root nitrogen concentration (RN).

	Phylogenetic generalized least squares					Ordinary least squares			
	RTD			RN		RTD		RN	
	df	F	*p*	F	*p*	F	*p*	F	*p*
Intercept	1	57.89	<.0001	48.23	<.0001	2042.49	<.0001	4403.27	<.0001
Diameter	1	49.69	<.0001	1.39	0.24	72.08	<.0001	6.44	0.011
Growth form	1	1.37	0.24	18.27	<.0001	1.08	0.3	56.40	<.0001
Mycorrhizal type	3	2.64	0.049	3.11	0.026	4.65	0.0032	6.31	0.0003
Diameter × Growth form	1	12.37	0.0005	1.19	0.28	8.76	0.0032	5.92	0.015
Diameter × Mycorrhizal type	3	5.95	0.0005	2.31	0.076	5.38	0.0012	3.77	0.011

Supplementary Table 2 Results of phylogenetic generalized least squares and ordinary least squares analyses for the effects of root diameter (Diameter) and mycorrhizal type (AM, EM and ERM) on root tissue density (RTD) and root nitrogen concentration (RN) in non-woody species.

	Phylogenetic generalized least squares					Ordinary least squares			
	RTD			RN		RTD		RN	
	df	F	*p*	F	*p*	F	*p*	F	*p*
Intercept	1	9.64	0.0021	69.97	<.0001	467.90	<.0001	1046.66	<.0001
Diameter	1	0.22	0.64	3.60	0.059	1.06	0.30	1.86	0.18
Mycorrhizal type	1	0.40	0.53	1.14	0.29	0.73	0.39	1.45	0.23
Diameter × Mycorrhizal type	1	0.46	0.50	<.001	0.99	0.022	0.88	0.41	0.52

Supplementary Table 3 Results of phylogenetic generalized least squares and ordinary least squares analyses for the effects of root diameter (Diameter) and mycorrhizal type (AM, EM and ERM) on root tissue density (RTD) and root nitrogen concentration (RN) in woody species

	Phylogenetic generalized least squares					Ordinary least squares			
	RTD			RN		RTD		RN	
	df	F	*p*	F	*p*	F	*p*	F	*p*
Intercept	1	188.54	<.0001	47.86	<.0001	1944.74	<.0001	3480.589	<.0001
Diameter	1	110.76	<.0001	0.0032	0.96	123.97	<.0001	14.17	0.0002
Mycorrhizal type	3	2.95	0.033	2.44	0.064	4.70	0.0031	4.79	0.0027
Diameter × Mycorrhizal type	3	9.01	<.0001	2.91	0.035	8.30	<.0001	4.37	0.0049

Supplementary Table 4 Results from linear mixed models testing the effects of data sources, root sampling and climatic zone on root tissue density (RTD) and root N concentration (RN).

		F	*p*
RTD	Root diameter	4.02	*
	Data source	5.21	*
	Root sampling	0.20	ns
	Climatic zone	0.90	ns
	Root diameter × Data source	13.60	***
	Root diameter × Root sampling	0.36	ns
RN	Root diameter	9.02	**
	Data source	0.25	ns
	Root sampling	12.76	**
	Climatic zone	2.04	ns
	Root diameter × Data source	1.97	ns
	Root diameter × Root sampling	0.53	ns

***, ** and * indicate significant levels at $p < 0.001$, $p < 0.01$, and $p < 0.05$, ns indicates not significant ($p > 0.05$). See Supplementary Data 1 for the definition of data source, root sampling and climatic zone; also see Introduction of the main text for details of the linear mixed models.

Supplementary References

1. Chen HYH, Brassard BW. Intrinsic and extrinsic controls of fine root life span. *Critical Reviews in Plant Sciences* **32**, 151-161 (2013).

2. Kong D*, et al.* Leading dimensions in absorptive root trait variation across 96 subtropical forest species. *New Phytologist* **203**, 863-872 (2014).

3. Xu Y. Fine root morphology, anatomy and tissue nitrogen and carbon of the first five order roots in twenty seven Chinese tropical hardwood tree species. Northeast Forestry University, China (2011).

4. Liu B, He J, Zeng F, Lei J, Arndt SK. Life span and structure of ephemeral root modules of different functional groups from a desert system. *New Phytologist* **211**, 103–112 (2016).

5. Valverde-Barrantes OJ, Horning AL, Smemo KA, Blackwood CB. Phylogenetically structured traits in root systems influence arbuscular mycorrhizal colonization in woody angiosperms. *Plant and Soil* **404**, 1-12 (2016).

6. McCormack ML, Adams TS, Smithwick EAH, Eissenstat DM. Predicting fine root lifespan from plant functional traits in temperate trees. *New Phytologist* **195**, 823-831 (2012).

7. Withington JM, Reich PB, Oleksyn J, Eissenstat DM. Comparisons of structure and life span in roots and leaves among temperate trees. *Ecological Monographs* **76**, 381-397 (2006).

8. Eissenstat DM, Wells CE, Yanai RD, Whitbeck JL. Building roots in a changing environment: implications for root longevity. *New Phytologist* **147**, 33-42 (2000).

9. Yu SQ*, et al.* Estimating fine-root longevity of *Fraxinux Mandshurica* and *Larix Gmelinii* using mini-rhizotrons. *Journal of Plant Ecology (Chinese Version)* **31**, 102-109 (2007).

10. Adams TS, McCormack ML, Eissenstat DM. Foraging strategies in trees of different root morphology: the role of root lifespan. *Tree physiology* **33**, 940-948 (2013).

11. Guo D, Mitchell RJ, Withington JM, Fan P-P, Hendricks JJ. Endogenous and exogenous controls of root life span, mortality and nitrogen flux in a longleaf pine forest: root branch order predominates. *Journal of Ecology* **96**, 737-745 (2008).

12. Assefa D, Godbold DL, Belay B, Abiyu A, Rewald B. Fine root morphology, biochemistry and litter quality indices of fast- and slow-growing woody species in the Ethiopian highland forests. *Ecosystems* **21**, 482-494 (2017).

13. Kong D, *et al.* Economic strategies of plant absorptive roots vary with root diameter. *Biogeosciences* **13**, 415-424 (2016).

14. Valverde-Barrantes OJ, Smemo KA, Blackwood CB, Norden N. Fine root morphology is phylogenetically structured, but nitrogen is related to the plant economics spectrum in temperate trees. *Functional Ecology* **29**, 796-807 (2015).
