# Supplementary material for: Nonlinearity of root trait relationships and the root economics spectrum
Source: Nat Commun. 2019 May 17;10:2203. doi: 10.1038/s41467-019-10245-6 (PMC6525182; doi:10.1038/s41467-019-10245-6)
Supplement: Supplementary file 3 — Reporting Summary [file 41467_2019_10245_MOESM3_ESM.pdf]

nature research

Corresponding author(s): Deliang Kong, Junjian Wang, Yulong Feng

Last updated by author(s): Apr 1, 2019

Reporting Summary

Nature Research wishes to improve the reproducibility of the work that we publish. This form provides structure for consistency and transparency in reporting. For further information on Nature Research policies, see Authors & Referees and the Editorial Policy Checklist .

Statistics

For all statistical analyses, confirm that the following items are present in the figure legend, table legend, main text, or Methods section.

n/a	Confirmed	
☐	☒	The exact sample size (*n*) for each experimental group/condition, given as a discrete number and unit of measurement
☒	☐	A statement on whether measurements were taken from distinct samples or whether the same sample was measured repeatedly
☐	☒	The statistical test(s) used AND whether they are one- or two-sided<br>*Only common tests should be described solely by name; describe more complex techniques in the Methods section.*
☐	☒	A description of all covariates tested
☐	☒	A description of any assumptions or corrections, such as tests of normality and adjustment for multiple comparisons
☐	☒	A full description of the statistical parameters including central tendency (e.g. means) or other basic estimates (e.g. regression coefficient) AND variation (e.g. standard deviation) or associated estimates of uncertainty (e.g. confidence intervals)
☒	☐	For null hypothesis testing, the test statistic (e.g. *F*, *t*, *r*) with confidence intervals, effect sizes, degrees of freedom and *P* value noted<br>*Give P values as exact values whenever suitable.*
☒	☐	For Bayesian analysis, information on the choice of priors and Markov chain Monte Carlo settings
☒	☐	For hierarchical and complex designs, identification of the appropriate level for tests and full reporting of outcomes
☒	☐	Estimates of effect sizes (e.g. Cohen's *d*, Pearson's *r*), indicating how they were calculated

Our web collection on statistics for biologists contains articles on many of the points above.

Software and code

Policy information about availability of computer code

Data collection	SigmaScan Pro software (V5.0, SPSS Inc., Chicago, USA)
Data analysis	SMATR: Standardised Major Axis Tests & Routines, Version 2.0; R software (v.3.30, R Core Team, 2016)

For manuscripts utilizing custom algorithms or software that are central to the research but not yet described in published literature, software must be made available to editors/reviewers. We strongly encourage code deposition in a community repository (e.g. GitHub). See the Nature Research guidelines for submitting code & software for further information.

Data

Policy information about availability of data

All manuscripts must include a data availability statement. This statement should provide the following information, where applicable:

- Accession codes, unique identifiers, or web links for publicly available datasets
- A list of figures that have associated raw data
- A description of any restrictions on data availability

The data used in this study can be found in the studies listed in the Supplementary Data 1 and the Supplementary References affiliated to the Supplementary figures, and are available upon request to the corresponding authors. The source data underlying the Supplementary Figure 4c are provided as a Source Data file, and are deposited in the Dryad Digital Repository: http://dx.doi.org/10.5061/dryad.sv2j191.

Field-specific reporting

Please select the one below that is the best fit for your research. If you are not sure, read the appropriate sections before making your selection.

Ecological, evolutionary & environmental sciences study design

All studies must disclose on these points even when the disclosure is negative.

Study description	Using over 800 plant species, we found in woody species (but not in non-woody species) nonlinear relationships between root diameter, and RTD and RN, which stemmed from the allometric relationship between stele and cortical tissues. The nonlinearity could potentially renew our knowledge on the root economics spectrum, and importantly, improve our understanding of the ecology, physiology and evolution of absorptive roots.
Research sample	We used the existing datasets on plant absorptive roots from published papers and theses. Details for the source of these datasets can refer to the Supplementary Data 1 in our study.
Sampling strategy	We collected plant species globally from published papers and theses. The sample size is sufficient as the number of species could be the largest by bar for studies exploring root trait relationships.
Data collection	We collected root trait data from tables and figures in the published papers and theses. D.K., J.W., H.W., H.Z. and Y.F. chose the studies and recorded the data in these studies into excel files for analyses.
Timing and spatial scale	We used data on absorptive root traits from studies from 2001 to 2018. Plant species in our study were collected globally.
Data exclusions	No data were excluded from the analyses.
Reproducibility	First, collect the data of absorptive root trait values along with plant phylogeny and other necessary information (e.g., growth forms, mycorrhizal types, climatic zones etc.) used in our analyses. Then, make average of root trait values for species appearing in different studies. Then, conduct the following analyses: (1) examine the allometric relationship between the steles and the ToS separately in woody and non-woody species. (2), explore the nonlinear relationships of RTD and RN with root diameter as predicted from allometric root anatomical structures. (3), perform the analyses regarding plant phylogeny, e.g., phylogenetic independent contrasts (PICs), phylogenetic signals (Blomberg's K and Pagel's λ), phylogenetic generalized least squares and ordinary least squares. (4), test the effects of the data source on the relationship between root diameter and RTD using a linear mixed model. The categories for the data source can refer to the Method section and the Supplementary Data 1.
Randomization	In the linear mixed model, the study was not considered as a random factor because they were not classified randomly but assigned to one of the data sources according to their trait relationships.
Blinding	The data used in our study were from published papers and theses that are accessible by referring to the studies listed in the Supplementary Data 1 and the Supplementary References affiliated to the Supplementary figures, and are available upon request to the corresponding authors.

Did the study involve field work? ☐ Yes ☒ No

Reporting for specific materials, systems and methods

We require information from authors about some types of materials, experimental systems and methods used in many studies. Here, indicate whether each material, system or method listed is relevant to your study. If you are not sure if a list item applies to your research, read the appropriate section before selecting a response.

Materials & experimental systems

n/a	Involved in the study	
☒	☐	Antibodies
☒	☐	Eukaryotic cell lines
☒	☐	Palaeontology
☒	☐	Animals and other organisms
☒	☐	Human research participants
☒	☐	Clinical data

Methods

n/a	Involved in the study	
☒	☐	ChIP-seq
☒	☐	Flow cytometry
☒	☐	MRI-based neuroimaging
