# Supplementary material for: Nonlinearity of root trait relationships and the root economics spectrum
Source: Nat Commun. 2019 May 17;10:2203. doi: 10.1038/s41467-019-10245-6 (PMC6525182; doi:10.1038/s41467-019-10245-6)
Supplement: Supplementary file 4 — Description of Additional Supplementary Files [file 41467_2019_10245_MOESM4_ESM.pdf]

Description of Additional Supplementary Files

Supplementary Data 1 Overview of the studies used in the analyses.

Supplementary Data 2 Regressions among root diameter (RD), thickness of root tissues outside the stele (tToS), the proportion of root cross sectional area occupied by the stele (PRS), root stele radius (SR), root tissue density (RTD) and root N concentration (RN) for figures presented in this study.
